# Influence of CO_2_ on neurovascular coupling: interaction with dynamic cerebral autoregulation and cerebrovascular reactivity

**DOI:** 10.1002/phy2.280

**Published:** 2014-03-26

**Authors:** Paola Maggio, Angela S. M. Salinet, Thompson G. Robinson, Ronney B. Panerai

**Affiliations:** 1 Neurologia Clinica, Università Campus Bio‐Medico, Rome, Italy; 2 Department of Cardiovascular Sciences, University of Leicester, Leicester, LE1 5WW, UK; 3 National Institutes for Health Research (NIHR), Biomedical Research Unit in Cardiovascular Sciences, Clinical Sciences Wing, Glenfield Hospital, Leicester, LE3 9QP, UK

**Keywords:** Carbon dioxide, cerebral hemodynamics, cerebrovascular reactivity, dynamic cerebral autoregulation, neurovascular coupling

## Abstract

PaCO_2_ affects cerebral blood flow (CBF) and its regulatory mechanisms, but the interaction between neurovascular coupling (NVC), cerebral autoregulation (CA), and cerebrovascular reactivity to CO_2_ (CVR), in response to hypercapnia, is not known. Recordings of cerebral blood flow velocity (CBFv), blood pressure (BP), heart rate, and end‐tidal CO_2_ (EtCO_2_) were performed in 18 subjects during normocapnia and 5% CO_2_ inhalation while performing a passive motor paradigm. Together with BP and EtCO_2_, a gate signal to represent the effect of stimulation was used as input to a multivariate autoregressive‐moving average model to calculate their separate effects on CBFv. Hypercapnia led to a depression of dynamic CA at rest and during stimulation in both hemispheres (*P *<**0.02) as well as impairment of the NVC response, particularly in the ipsilateral hemisphere (*P *<**0.01). Neither hypercapnia nor the passive motor stimulation influenced CVR. Dynamic CA was not influenced by the motor paradigm during normocapnia. The CBFv step responses to each individual input (BP, EtCO_2_, stimulation) allowed identification of the influences of hypercapnia and neuromotor stimulation on CA, CVR, and NVC, which have not been previously described, and also confirmed the depressing effects of hypercapnia on CA and NVC. The stability of CVR during these maneuvers and the lack of influence of stimulation on dynamic CA are novel findings which deserve further investigation. Dynamic multivariate modeling can identify the complex interplay between different CBF regulatory mechanisms and should be recommended for studies involving similar interactions, such as the effects of exercise or posture on cerebral hemodynamics.

## Abstract (precis)

The influence of hypercapnia on dynamic cerebral autoregulation (CA), CO_2_ vasoreactivity (CVR), and neurovascular coupling (NVC) was described based on a single recording during motor stimulation coupled to a new multivariate modeling approach. Hypercapnia led to a depression of CA at rest and during stimulation in both hemispheres as well as impairment of the NVC response. Neither hypercapnia nor the passive motor stimulation influenced CVR. Dynamic CA was not influenced by the motor paradigm during normocapnia. The stability of CVR during these maneuvers and the lack of influence of stimulation on dynamic CA are novel findings which deserve further investigation.

## Introduction

Neural activation, induced by cognitive or sensorimotor paradigms, leads to increases in cerebral blood flow (CBF), a mechanism known as *neurovascular coupling* (NVC) (Girouard and Iadecola 2006). Quantification of the NVC response to stimulation is commonly performed by assessing the percent change from baseline values in CBF or CBF velocity (CBFv), recorded with transcranial Doppler ultrasound (TCD) or functional MRI, without taking into account the potential influence of other noncerebral hemodynamic variables. More recently, the limitations of this approach have become clear with the demonstration that the CBF/CBFv response is significantly influenced by concomitant changes in arterial blood pressure (BP) and PaCO_2_ (Moody et al. 2005; Panerai et al. 2012b; Salinet et al. 2012a).

These observations have considerable implications for a better understanding of physiological and clinical studies of NVC. Changes in BP induced by the maneuver will trigger an autoregulatory response, contributing to further transient changes in CBFv. As shown by Aaslid and coworkers (Aaslid et al. 1989; Tiecks et al. 1995), rapid changes in BP are followed by similar changes in CBFv, but due to the mechanism of dynamic *cerebral autoregulation* (CA), CBFv will return to its baseline value within a few seconds, even if BP remains displaced from baseline values. Moreover, changes in PaCO_2_, often estimated from continuous recordings of end‐tidal CO_2_ (EtCO_2_), will also lead to changes in CBF due to the well‐known mechanism of *cerebrovascular reactivity* to CO_2_ (CVR) (Poulin et al. 1996; Brian 1998; Vernieri et al. 1999; Battisti‐Charbonney et al. 2011; Willie et al. 2012).

In a previous study, we have shown that hypercapnia impairs the CBFv response to passive motor stimulation (Maggio et al. 2013). Analysis of instantaneous pressure–velocity curves indicated that the main effects resulted from a reduced critical closing pressure (CrCP) response, thus suggesting an impairment of metabolic control (Panerai et al. 2005, 2012a; Maggio et al. 2013) On the other hand, resistance‐area product (RAP), which could be an indicator of myogenic control (Panerai et al. 2005), was not affected by hypercapnia, thus raising doubts about the influence of hypercapnia on dynamic CA during neural stimulation. To compare the efficacy of dynamic CA at rest and during neural stimulation, at both normocapnia and hypercapnia, using conventional approaches, such as transfer function analysis (Zhang et al. 1998; Panerai et al. 1999), is technically complex due to the nonstationarity induced by motor stimulation and also for neglecting the dynamic contribution of fluctuations in PaCO_2_. To overcome this difficulty, multivariate models have been proposed using as input variables both fluctuations in BP and EtCO_2_ (Panerai et al. 2000; Edwards et al. 2004; Mitsis et al. 2006). Using a similar approach, we have reanalyzed the same set of data in (Maggio et al. 2013) using a more elaborate model recently described (Panerai et al. 2012b) which can provide simultaneous estimates of dynamic CA and CVR at rest, together with estimates of NVC during motor stimulation. Our objective was to test the hypothesis that all three CBF regulatory mechanisms (CA, CVR, NVC) are impaired by mild hypercapnia.

## Materials and Methods

### Subjects and measurements

This study was based in the same set of data collected by Maggio et al. (2013). Healthy right‐handed middle‐aged volunteers (>45 years old) were recruited from departmental staff and their relatives. Right‐handedness was assessed with the Edinburgh inventory (Oldfield 1971). Exclusion criteria included physical disease in the upper limb, poor insonation of both temporal bone windows, and any history of cardiovascular, neurological, or respiratory disease. The Nottingham Research Ethics Committee 1, United Kingdom (Ref: 11/EM/0016), approved the study and informed written consent was obtained from all participants.

Volunteers avoided caffeine, alcohol, and nicotine for ≥12 h before attending a quiet laboratory with constant ambient temperature of 24°C. Beat‐to‐beat BP was recorded continuously using the Finometer^®^ device (FMS, Finapres Measurement Systems, Arnhem, Netherlands), attached to the middle finger of the left hand. The servo‐correcting mechanism of the Finometer^®^ was switched on and then off prior to measurements. Heart rate (HR) interval was recorded using a 3‐lead electrocardiogram (ECG) and EtCO_2_ was measured via nasal prongs (Salter Labs) by a capnograph (Capnocheck Plus). Bilateral insonation of the middle cerebral arteries (MCAs) was performed using TCD (Viasys Companion III; Viasys Healthcare) with a 2 MHz probe, which was secured in place using a head‐frame. Hypercapnia was induced by the inhalation of a mixture of 5% CO_2_/air through a mask. During CO_2_ breathing, the capnograph was connected to the mask via a sample line. During the entire procedure, subjects were in a supine position and detailed instructions were given before taking measurements.

After a period of 15‐min stabilization, participants performed a 5‐min baseline recording and two passive motor paradigms during air breathing. The same sequence was repeated during CO_2_ inhalation. Arterial BP was measured with a sphygmomanometer before each measurement.

The paradigm was performed only with the dominant arm and consisted of an examiner performing repetitive flexion and extension of the subject's elbow within a range of movement of approximately 90° at a rate of 1 Hz, given by the sound of a metronome. Subjects were instructed to relax and not move the arm. All paradigm recordings started with a 90 sec baseline phase. Thereafter, the paradigm was performed over 60 sec, with a 90 sec recovery phase. During the rest and recovery periods, the examiner kept hold of the participant's arm.

### Data analysis

Data were simultaneously recorded onto a data acquisition system (PHYSIDAS, Department of Medical Physics, University Hospitals of Leicester) for subsequent off‐line analysis. ECG, EtCO_2_, BP, and stimulus marker signals were sampled at 500 samples s^−1^, and BP was calibrated at the start of each recording. All signals were visually inspected to identify artefacts and noise, and narrow spikes (<100 ms) were removed by linear interpolation. The CBFv channels were subjected to a median filter and all signals were low‐pass filtered with a cut‐off frequency of 20 Hz. The R–R interval was then automatically marked from the ECG and continuous HR plotted against time. Occasional ectopic beats caused spikes in the HR signal; these were manually removed by remarking the R–R intervals for the time points at which they occurred. Mean BP and CBFv values were calculated for each cardiac cycle. The end of each expiratory phase was detected in the EtCO_2_ signal, linearly interpolated, and resampled with each cardiac cycle. Beat‐to‐beat data were spline interpolated and resampled at 5 samples s^−1^ to produce signals with a uniform time‐base. With the use of the electrical output from the metronome, the stimulation signal *s(t)* was added to the ensemble. The maneuver that achieved the highest amplitude of contralateral CBFv response was chosen to represent the participant's response (Salinet et al. 2012b).

A multivariate autoregressive‐moving average (ARMA) model was used to represent the dynamic influence of BP, EtCO_2_, and stimulus [*s(t)*] (inputs) on CBFv (output). These time‐domain models have been used previously to analyze the simultaneous effects of BP and PaCO_2_ on CBFv (Panerai et al. 2000; Edwards et al. 2004; Mitsis et al. 2004, 2006). More recently, we have extended their application to NVC by adding the effects of stimulation through the *s(t)* input (Peebles et al. 2012; Salinet et al. 2012b). The order of these models, representing the number of past samples adopted for the autoregressive (AR) and moving average (MA) terms, was thoroughly considered (Peebles et al. 2012). As described in detail elsewhere (Peebles et al. 2012; Salinet et al. 2012b), ARMA coefficients were used to estimate the CBFv step response to each individual input which then represents the efficacy of the three separate regulatory mechanisms, that is CA, CVR, and NVC. Using the model autoregressive and moving average coefficients, it is possible to obtain the CBFv step response to each of the separate inputs. Step responses for each subject were averaged to obtain the population mean and SD step responses. For the CBFv step response for the BP input, which represents dynamic CA (Panerai et al. 2012b), the autoregulation index (ARI) proposed by Tiecks et al. (1995) was extracted by using the best least‐squares fit between the CBFv step response and one of the 10 model ARI curves proposed by Tiecks et al. (1995). ARI was computed for each subject separately for left and right hemisphere during normocapnia and hypercapnia. The CBFv step responses to the EtCO_2_ and *s(t)* inputs were quantified by their change from baseline to the plateau region, defined as the mean value of the response from 20–25 sec, expressed by parameters ΔS_CO2_ and ΔS_*s*(*t*)_, respectively. ΔS_CO2_ is a measure of CVR to CO_2_. During baseline, the same multivariate model was adopted to estimate the CBFv step responses to the BP and EtCO_2_ inputs, by setting the third input (*s(t)*) to zero and corresponding values of ARI and ΔS_CO2_ were computed as for the stimulation phase.

### Statistical analysis

Mean values of each variable were extracted from the 30 sec phase before the start of the paradigm to reflect baseline conditions. Student's *T*‐test for dependent variables was used to compare baseline values of CBFv, BP, HR, EtCO_2_, and ARI between normocapnia and hypercapnia.

To compare changes in CBFv between normocapnia and hypercapnia, the area‐under‐the‐curve (AUC) was calculated for their differences from the beginning of the maneuver, up to 20 sec after the end of passive arm movement. Statistical analysis was performed using two‐way repeated measures ANOVA with type (normocapnia vs. hypercapnia) and side (contralateral vs. ipsilateral) as within factors for CBFv variations. Two‐way repeated measures ANOVA was also adopted to test for differences in ARI and ΔS_CO2_ due to hypercapnia and the effect of the maneuver in comparison to baseline values. When appropriate, a post hoc test for multiple comparisons (Tukey's test) was performed. A value of *P *<**0.05 was adopted to indicate statistical significance. All statistical analyses were performed with *Statistica*^®^ software for Windows.

## Results

Nineteen participants (eight male) of mean age 58.1 years (SD 7.1, range 48–77) were recruited. Participant's mean Edinburgh Inventory for right‐handedness was 96.5% (8.5). All participants completed normocapnia measurements. Three participants performed the baseline recording and only one passive motor maneuver with 5% CO_2_ due to intolerance to the mask. In these cases, we analyzed the only available passive motor paradigm recording to represent the participant's response.

As described previously (Maggio et al. 2013), hypercapnia increased CBFv from 56.6 (11.8) to 72.0 (14.8) cm s^−1^ (*P *<**0.001), in the contralateral MCA, and from 49.4 (9.1) to 62.2 (11.2) cm s^−1^ (*P *<**0.001) in the ipsilateral MCA. Mean BP increased from 99.4 (11.1) to 109.1 (19.3) mmHg (*P *<**0.01) and EtCO_2_ increased from 40.7 (3.3) to 47.9 (3.2) mmHg (*P *<**0.001).

### CBFv responses and its contributors

One subject was excluded from further analysis, as he did not show a CBFv response to the paradigm during normocapnia.

**Figure 1.**
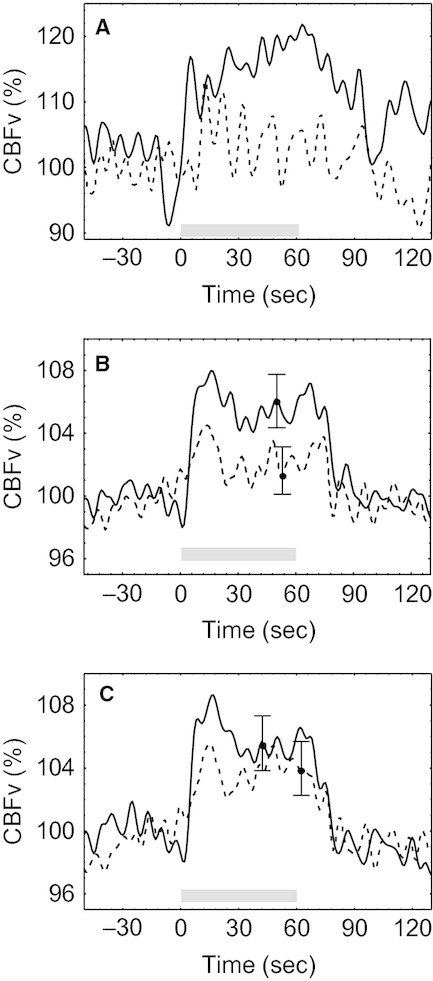
(A) Representative CBFv response from a single subject during normocapnia (continuous line) and hypercapnia (dashed line) to repetitive elbow flexion from 0– 60 sec (gray bar). Population averages of CBFv responses for the ipsilateral (B) and contralateral (C) middle cerebral arteries. Error bars represent the largest ±1 SE.

In both normocapnic and hypercapnic conditions, CBFv increased bilaterally with the beginning of passive movement and showed a plateau phase that outlasted the duration of stimulation (Fig. 1). This characteristic bilateral rise in CBFv (AUC 105.2 (4.6) % ipsilateral; 104.9 (3.8) % contralateral) was significantly reduced by hypercapnia (AUC 102.2 (3.0) % ipsilateral; 103.2 (2.9) % contralateral, ANOVA, *P *=**0.01). A post hoc comparison indicated a significant difference for the ipsilateral hemisphere only (Tukey, *P *=**0.001).

**Figure 2.**
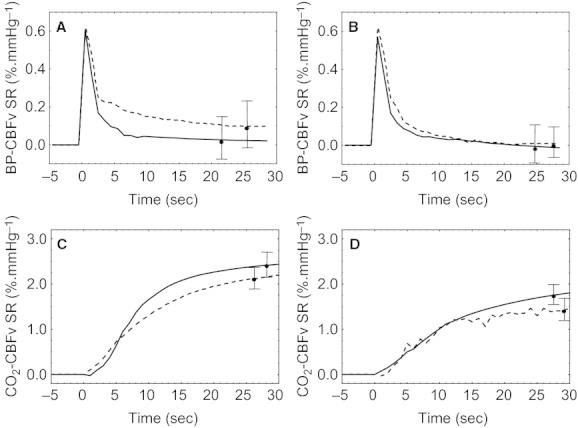
Population average CBFv step responses (SR) due to BP (A, B) and EtCO_2_ (C, D) at baseline. Time *t *=**0 represent the beginning to the CBFv SR to each of the two inputs. Separate responses given for the ipsilateral (A, C) and contralateral (B, D) MCA during normocapnia (continuous line) and hypercapnia (dashed line). Error bars represent the largest ±1 SE.

**Figure 3.**
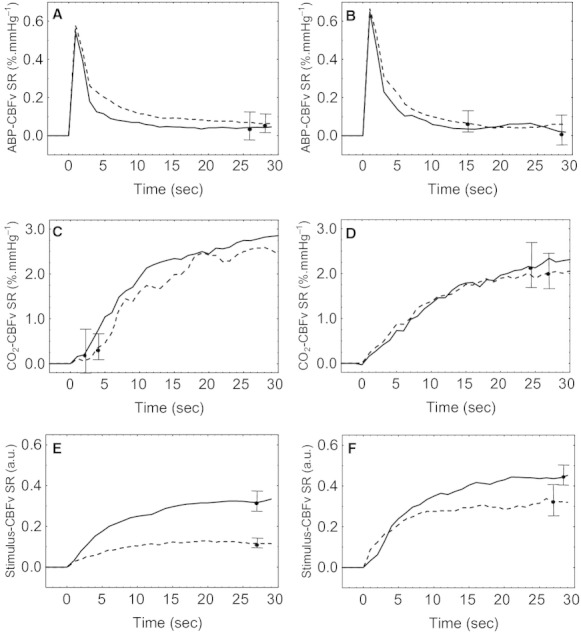
Population average CBFv step responses (SR) during passive elbow flexion due to BP (A, B), EtCO_2_ (C, D), and motor stimulation (E, F). Time *t *=**0 represent the beginning to the CBFv SR to each of the three inputs. Separate responses given for the ipsilateral (A, C, E) and contralateral (B, D, F) MCA during normocapnia (continuous line) and hypercapnia (dashed line). Error bars represent the largest ±1 SE.

Bilateral CBFv step responses to the BP input extracted during baseline and motor stimulation are represented in Figs. 2A,B and 3A,B, respectively. From these step responses, ARI was calculated during normocapnia and hypercapnia. At baseline, ARI values decreased during hypercapnia from 6.1 (1.0) to 4.8 (1.1) (*P *<**0.002) in the ipsilateral hemisphere, and from 6.1(1.3) to 4.8 (1.9) (*P *<**0.01) in the contralateral hemisphere. Two‐way ANOVA revealed significant differences between groups (*F *=**4.1, *P *=**0.001), and the post hoc Tukey test showed a significant decrease in ARI values during hypercapnia (*P *=**0.002 and 0.01 for the contralateral and ipsilateral hemispheres, respectively). During stimulation, the mean ARI values decreased from 6.1 (0.7) in the contralateral hemisphere and 6.1 (1.2) in the ipsilateral during normocapnia to 5.0 (1.6) and 5.2 (1.2) during hypercapnia, indicating a significant impairment in CA (Tukey *P *=**0.005 and *P *=**0.02 for the contralateral and ipsilateral hemispheres, respectively). The similarity between these values and the corresponding ARI during baseline was confirmed by the result of the two‐way ANOVA showing that the passive motor maneuver (during normocapnia) had no significant effect on dynamic CA.

Corresponding step responses for the EtCO_2_ (during baseline and stimulation) and motor stimulus inputs showed similar patterns, rising gradually to reach a plateau within 15 sec (Figs. 2C,D and 3C,D). The CBFv step response to CO_2_ and the ΔS_CO2_ parameter were not significantly changed by either hypercapnia or passive elbow flexion. At baseline, the mean values of ΔS_CO2_ were 2.1 (1.3) cm s^−1^ mmHg^−1^ and 1.5 (1.6) cm s^−1^ mmHg^−1^ for the ipsilateral hemisphere, and 1.4 (1.8) cm s^−1^ mmHg^−1^ and 0.8 (1.7) cm s^−1^ mmHg^−1^ for the contralateral hemisphere during normocapnia and hypercapnia, respectively. During stimulation, ΔS_CO2_ was 2.8 (1.6) cm s^−1^ mmHg^−1^ and 2.6 (0.8) cm s^−1^ mmHg^−1^ ipsilateral and 1.6 (2.0) cm s^−1^ mmHg^−1^ and 1.4 (1.9) cm s^−1^ mmHg^−1^ contralateral, again for normocapnia and hypercapnia, respectively. For the CBFv step responses to the stimulus input (Fig. 3E,F), ΔS_*s*(*t*)_ varied from 0.3 (0.09) (A.U.) and 0.4 (0.08) during normocapnia to 0.09 (0.03) (*P *=**0.001) and 0.1 (1.0) (*P *=**0.04) during hypercapnia for the ipsilateral and contralateral hemispheres, respectively.

## Discussion

### Main findings

To our knowledge, this is the first study to model the simultaneous influences of hypercapnia on NVC, dynamic CA, and CVR. Our results confirmed that dynamic CA was impaired by hypercapnia both at rest and during neural stimulation, and that NVC was also impaired by breathing 5% CO_2_ in air. On the other hand, CVR was not impaired by hypercapnia or neural stimulation and dynamic CA was not affected by passive elbow flexion during normocapnia. The CBFv step responses to each input (BP, EtCO_2_, *s(t)*) had temporal patterns compatible with previous studies. For the BP input, the CBFv step response showed the classical rapid rise, followed by a slower decline as expected of a working dynamic CA (Tiecks et al. 1995; Panerai et al. 2001). As expected, hypercapnia shifted the response upwards (Panerai et al. 1999) and reduced the value of the ARI parameter (Katsogridakis et al. 2013). Of considerable interest, this response was not influenced by passive motor stimulation itself.

The CBFv step response to the EtCO_2_ input had a temporal pattern showing a gradual rise as obtained in direct measurements of step changes in PaCO_2_ (Poulin et al. 1996) as well as in previous studies based on system identification techniques (Edwards et al. 2004; Mitsis et al. 2004; Salinet et al. 2012a). The ΔS_CO2_ parameter is equivalent to the classical assessment of CVR using the difference in CBFv from baseline to a steady‐state value after breathing a CO_2_‐rich mixture with air. This parameter was in very good agreement with corresponding values of CVR published in the literature (Ide et al. 2003; Moody et al. 2005; Willie et al. 2012), and it was not significantly influenced by hypercapnia during baseline or by passive motor stimulation. The third step response, that quantifies the influence of stimulation on CBFv, also had a gradual rise to a plateau which was significantly reduced by hypercapnia, as shown by the complete step response curves (Fig. 3), as well as by the ΔS_*s*(*t*)_ parameter. These curves are in excellent agreement with results obtained from a different group of healthy subjects (Panerai et al. 2012b), but their sensitivity to PaCO_2_ had not been reported previously.

### Complexity of the NVC response to elbow flexion

One important limitation of NVC studies based on imaging techniques is the difficulty of recording simultaneous changes in BP and other variables that are also disturbed by cognitive or sensorimotor paradigms. As a result, most imaging studies tend to ascribe all changes in CBF to the stimulus when in fact there is substantial contribution from peripheral determinants as shown by previous studies (Moody et al. 2005; Panerai et al. 2005, 2012b; Salinet et al. 2012a,b, 2013a,b; Maggio et al. 2013; Nogueira et al. 2013). These results cannot be generalized though as different paradigms are likely to have variable degrees of influence on BP and autonomic nervous system control, as well as on respiratory frequency and other physiological covariates (Moody et al. 2005; Salinet et al. 2012a). For this reason, studies of NVC should ideally include simultaneous measurements of BP, EtCO_2_, and other physiological variables that could influence the CBFv response under different circumstances. Due to parallel changes in BP and PaCO_2_, a clear interaction exists between dynamic CA, CVR, and NVC. This raises the question of whether multivariate dynamic modeling should be the tool of choice in the context of NVC studies, to allow for simultaneous assessment of dynamic CA and CVR.

Changes in BP during CO_2_ stimuli have direct effects on CBFv (Claassen et al. 2007), so that only in the CBFv response range below the threshold for the increase in BP with CO_2_ the CBFv measurement reflect vascular reactivity to CO_2_ alone (Battisti‐Charbonney et al. 2011). Simultaneous modeling of the influences of BP and PaCO_2_ on CBFv have been described previously, mainly for recordings performed at rest (Peebles et al. 2012) but also for perturbations in PaCO_2_ (Edwards et al. 2004) and orthostatic challenges induced with lower body negative pressure (Mitsis et al. 2006). In particular, Edwards et al. (2004) have demonstrated the sensitivity of CBFv step responses to BP and EtCO_2_ for both hypo‐ and hypercapnia, and Mitsis et al. (2004) have identified a dynamic interaction between the BP and EtCO_2_ inputs. Nevertheless, none of these models have addressed the interaction between dynamic CA, CVR, and NVC mechanisms. One recent study though has introduced a technique to compensate for the influence of BP on NVC responses (Spronck et al. 2012), based on Tiecks et al. (1995) step response templates for the ARI parameter.

One important aspect of the interaction of different CBF regulatory mechanisms is whether CA and/or CVR are depressed during neural activation. Applying classical techniques of assessment, dynamic CA was found to be impaired during cognitive stimulation based on both word construction and a 2D puzzle, which preferentially activate the left and right hemispheres, respectively (Panerai et al. 2005). Similar conclusions could be drawn from other studies (Nakagawa et al. 2009; Dineen et al. 2010; Nogueira et al. 2013). The finding that the CA response was not altered by passive motor stimulation in the present study could be explained by differences in the intensity of the alert reaction induced by other paradigms. An alternative explanation though would be the fact that multivariate modeling takes into account the influences of PaCO_2_ and the stimulus, thus rendering more accurate estimates of dynamic CA. Similar concerns would apply to studies of cerebral hemodynamics during exercise, where CA was found to be normal during mild exercise but depressed during exhaustive exercise (Ogoh et al. 2005).

Unaccounted for interactions with BP and stimulation could also explain why the step responses to CO_2_ (Fig. 3) and the ΔS_CO2_ parameter did not show changes with hypercapnia per se or due to the *s(t)* input. The CBFv‐arterial CO_2_ relationship has been described as exponential by some authors (Markwalder et al. 1984; Ide et al. 2003), but others have reported a more linear relationship (Claassen et al. 2007; Willie et al. 2012), mainly in the range of 40–50 mmHg as was the case of our study. The latter would explain a constant CVR with increasing levels of PaCO_2_ as we have found. Moreover, the exponential relationship was established with steady‐state changes in PaCO_2_, and did not take into account interactions with BP (Dumville et al. 1998). However, using the two‐breath technique, Edwards et al. (2004) obtained step responses to EtCO_2_ changes that were depressed by hypercapnia. Those investigators took into account influences of BP by using cerebrovascular conductance, instead of CBFv, as the model output variable. The lack of a significant effect of hypercapnia on the step responses to CO_2_ in our case could be due to the much smaller signal‐to‐noise ratio of the EtCO_2_ input, resulting from spontaneous fluctuations, as compared to the relatively larger changes induced by the two‐breath technique with inhalation of 8% CO_2_.

The interaction of CVR with passive motor stimulation also deserves attention. The influence of sympathetic stimulation on classical measures of CVR is still controversial with studies showing both significant (Peebles et al. 2012) and nonsignificant influences (LeMarbre et al. 2003). An increase in CVR was reported during whole‐body exercise (Ogoh et al. 2005) and it is possible that the absence of an effect in our case was due to the much smaller muscle mass involved in passive elbow flexion.

Overall, the considerations above suggest that in the presence of physiological changes induced by cognitive/sensorimotor paradigms, posture, exercise, breathing, or other maneuvers, multivariate modeling should be the tool of choice. Nevertheless, further work in this area is needed to optimize the use of multivariate modeling in studies of cerebral hemodynamics.

### Influence of CO_2_ on NVC

The marked reduction in the NVC response induced by hypercapnia, as reflected by the CBFv step responses to the *s(t)* input (Fig. 3) and the ΔS_*s*(*t*)_ parameter, is in agreement with previous studies in humans and animals (Xu et al. 2011; Kennerley et al. 2012), but at odds with similar studies based on visual stimulation and CBFv measurements in the posterior cerebral artery (PCA). Rosengarten et al. (2003) found no differences due to hypercapnia in the CBFv response to a reading paradigm. On the other hand, using the same protocol, Szabo et al. (2011) reported a reduction in the CBFv response to visual stimulation in healthy young subjects during hypocapnia induced by hyperventilation. These apparently contradictory results might be explained by regional differences in NVC between the visual and motor cortex (Chiarelli et al. 2007). Although regional differences in CVR have been reported, based on MRI measurements (Ito et al. 2000), no differences in CVR were obtained comparing transcranial Doppler recordings of the MCA with those from the PCA (Willie et al. 2012). One important alternative possibility though is that the effects of changes in PaCO_2_ on NVC, and differences between motor and visual areas, are mainly related to the direct influence of CO_2_ on the neural response to stimulation, instead of the vasomotor effects resulting from changes in pH (Yoon et al. 2012). This interpretation could also explain the ‘flow paradox’ reflected by the baseline changes in CBFv when compared to the amplitude of the NVC response. In our study, hypercapnia increased baseline CBFv by more than 25% in both hemispheres. On the other hand, neural stimulation resulting from elbow flexion, led to an increase in CBFv of the order of 5% (Fig. 1). Therefore, during hypercapnia there would be enough excess blood flow to satisfy the metabolic demand of neural stimulation. Nevertheless, a response could still be detected, albeit of lesser amplitude (Fig. 1). These observations suggest that the flow response is directly linked to neural signaling and not to the feedback effect of increased demand for O_2_ and that it is the forward neural response that is affected by hypercapnia (Yoon et al. 2012). Further support for this interpretation can be derived from the study of Xu et al. (2011) in humans showing that hypercapnia has a suppressive effect on brain activity, including a reduction in functional connectivity. In rats, Kennerley et al. (2012) also demonstrated that the differences observed due to hypercapnia on the response to whisker stimulation were mainly reflecting changes in neural activity than in the demand for O_2_. The lack of effect of hypercapnia during visual stimulation (Rosengarten et al. 2003) suggests that the direct influence of CO_2_ on neural activity might be different in the visual cortex and could also explain the results of hypocapnia induced by hyperventilation (Szabo et al. 2011). More work in this area should be a priority, including studies of the effects of hyperventilation during sensorimotor paradigms.

### Limitations of the study

Changes in CBFv can only reflect corresponding changes in CBF if the diameter of the insonated artery remains constant, an assumption that has been supported by observational and imaging studies (Giller et al. 1993; Serrador et al. 2000). These studies did not show changes in MCA diameter due to hypercapnia, but similar investigations are lacking for the potential influence of neural stimulation. If MCA diameter should increase due to passive elbow flexion, our recorded changes in CBFv due to stimulation would be an underestimate of the true changes in CBF thus biasing our results in the direction of type II errors.

The multivariate model, based on ARMA structures is limited to a linear representation of input–output relationships. Although nonlinear models have been proposed for dynamic CA, their superiority has not been demonstrated (Panerai et al. 2001; Mitsis et al. 2004). Nevertheless, one aspect of multiple input nonlinear models is the possibility to incorporate input–input interactions, as demonstrated by Mitsis et al. (2004) for the BP and EtCO_2_ inputs. Further work is needed to explore the relevance of incorporating this type of interaction in the model, mainly from the perspective of improving the reliability of clinical applications.

Separate training and testing sets of data were not appropriate to the nature of this study as the main objective was not to propose a general model for CA, CVR, and NVC, but simply to use the flexibility of the ARMA structure to extract parameters leading to the estimation of CBFv step responses to each input. This is equivalent to the application of linear regressions to extract the slope of static relationships.

Motor stimulation with the dominant arm would be expected to lead to pronounced interhemispherical differences. In our study, contralateral CBFv step responses to stimulation (Fig. 1) were significantly greater in comparison with the ipsilateral side thus confirming the physiological consistency of the model. On the other hand, lateralization was somewhat limited, possibly due to the nature of the paradigm, involving more diffuse and nonspecific stimulation regarding attention, concentration, and motivation.

Finally, in our previous communication (Maggio et al. 2013), ARI values were smaller than in the current study. In both cases, ARI values were estimated by fitting Tiecks et al. (1995) template responses, but in our first study, CBFv step responses were obtained by FFT‐based transfer function analysis, while in the current study, step responses were estimated using ARMA models. In previous studies of dynamic CA, we also observed that mean normal values obtained with ARMA modeling were higher than those obtained with classical transfer function analysis (Panerai et al. 2003, 2010).

## Conclusions

Hypercapnia induces cerebral vasodilation and impairment of CBF regulatory mechanisms, chiefly CA. We confirmed that NVC was also impaired by breathing 5% CO_2_ in air, but CVR was not influenced either by hypercapnia or neural stimulation, and dynamic CA was not influenced by passive motor stimulation. The NVC response during hypercapnia can be better explained by a direct effect of CO_2_ on neural activity, rather than the vasodilation associated with reduced pH. More research is needed on the lack of change in CVR due to hypercapnia. Neural stimulation by cognitive or sensorimotor paradigms induces simultaneous changes in BP and PaCO_2_, thus requiring the use of multivariate dynamic models for identification of its separate influences and more robust assessment of the NVC response. Further application of this approach is warranted under diverse physiological and clinical conditions.

## Conflict of interest

None declared.

## References

[b1] Aaslid R. Lindegaard K. F. Sorteberg W. Nornes H. 1989 Cerebral auto‐regulation dynamics in humans. Stroke; 20:45-52. 2492126 10.1161/01.str.20.1.45

[b2] Battisti‐Charbonney A. Fisher J. Duffin J. 2011 The cerebrovascular response to carbon dioxide in humans. J. Physiol.; 589:3039-3048. 21521758 10.1113/jphysiol.2011.206052 PMC3139085

[b3] Brian J. E. J. 1998 Carbon dioxide and the cerebral circulation. Anesthesiology; 88:1365-1386. 9605698 10.1097/00000542-199805000-00029

[b4] Chiarelli P. A. Bulte D. P. Gallichan D. Piechnik S. K. Wise R. Jezzard P. 2007 Flow‐metabolism coupling in human visual, motor, and supplementary motor areas assessed by magnetic resonance imaging. Magn. Reson. Med.; 57:538-547. 17326178 10.1002/mrm.21171

[b5] Claassen J. A. Zhang R. Fu Q. Witkowski S. Levine B. D. 2007 Transcranial Doppler estimation of cerebral blood flow and cerebrovascular conductance during modified rebreathing. J. Appl. Physiol.; 102:870-877. 17110510 10.1152/japplphysiol.00906.2006

[b6] Dineen N. E. Brodie F. G. Robinson T. G. Panerai R. B. 2010 Continuous estimates of dynamic cerebral autoregulation during transient hypocapnia and hypercapnia. J. Appl. Physiol.; 108:604-613. 20035062 10.1152/japplphysiol.01157.2009 PMC2838633

[b7] Dumville J. Panerai R. B. Lennard N. S. Naylor A. R. Evans D. H. 1998 Can cerebrovascular reactivity be assessed without measuring blood pressure in patients with carotid artery disease? Stroke; 29:968-974. 9596244 10.1161/01.str.29.5.968

[b8] Edwards M. R. Devitt D. L. Hughson R. L. 2004 Two‐breath CO_2_ test detects altered dynamic cerebrovascular autoregulation and CO_2_ responsiveness with changes in arterial PCO_2_. Am. J. Physiol. Regul. Integr. Comp. Physiol.; 287:R627-R632. 15044183 10.1152/ajpregu.00384.2003

[b9] Giller C. A. Bowman G. Dyer H. Mootz L. Krippner W. 1993 Cerebral arterial diameters during changes in blood pressure and carbon dioxide during craniotomy. Neurosurgery; 27:737-741. 8492848

[b10] Girouard H. Iadecola C. 2006 Neurovascular coupling in the normal brain and in hypertension, stroke and Alzheimer disease. J. Appl. Physiol.; 100:328-335. 16357086 10.1152/japplphysiol.00966.2005

[b11] Ide K. Eliasziw M. Poulin M. J. 2003 Relationship between middle cerebral artery blood velocity and end‐tidal PCO_2_ in the hypocapnic‐hypercapnic range in humans. J. Appl. Physiol.; 95:129-137. 19278048 10.1152/japplphysiol.01186.2002

[b12] Ito H. Yokoyama I. Iida H. Kinoshita T. Hatazawa J. Shimosegawa E. 2000 Regional differences in cerebral vascular response to PaCO_2_ changes in humans measured by positron emission tomography. J. Cereb. Blood Flow Metab.; 20:1264-1270. 10950385 10.1097/00004647-200008000-00011

[b13] Katsogridakis E. Bush G. Fan L. Birch A. A. Simpson D. M. Allen R. 2013 Detection of impaired cerebral autoregulation improves by increasing arterial blood pressure variability. J. Cereb. Blood Flow Metab.; 33:519-523. 23232946 10.1038/jcbfm.2012.191 PMC3618385

[b14] Kennerley A. J. Harris S. Bruyns‐Haylett M. Boorman L. Zheng Y. Jones M. 2012 Early and late stimulus‐evoked cortical hemodynamic responses provide insight into the neurogenic nature of neurovascular coupling. J. Cereb. Blood Flow Metab.; 32:468-480. 22126914 10.1038/jcbfm.2011.163 PMC3293120

[b15] LeMarbre G. Stauber S. Khayat R. N. Puleo D. S. Skatrud J. B. Morgan B. J. 2003 Baroreflex‐induced sympathetic activation does not alter cerebrovascular CO_2_ responsiveness in humans. J. Physiol.; 551:609-616. 12844511 10.1113/jphysiol.2003.046987 PMC2343219

[b16] Maggio P. Salinet A. S. M. Panerai R. B. Robinson T. G. 2013 Does hypercapnia‐induced impairment of cerebral autoregulation affect neurovascular coupling? A functional TCD study. J. Appl. Physiol.; 115:491-497. 23743398 10.1152/japplphysiol.00327.2013 PMC3742941

[b17] Markwalder T.‐M. Grolimund P. Seiler R. W. Roth F. Aaslid R. 1984 Dependency of blood flow velocity in the middle cerebral artery on end‐tidal carbon dioxide partial pressure: a transcranial ultrasound doppler study. J. Cereb. Blood Flow Metab.; 4:368-372. 6432808 10.1038/jcbfm.1984.54

[b18] Mitsis G. D. Poulin M. J. Robbins P. A. Marmarelis V. Z. 2004 Nonlinear modeling of the dynamic effects of arterial pressure and CO_2_ variations on cerebral blood flow in healthy humans. IEEE Bio. Med. Eng.; 51:1932-1943. 10.1109/TBME.2004.834272 15536895

[b19] Mitsis G. D. Zhang R. Levine B. D. Marmarelis V. Z. 2006 Cerebral hemodynamics during orthostatic stress assessed by nonlinear modeling. J. Appl. Physiol.; 101:354-366. 16514006 10.1152/japplphysiol.00548.2005

[b20] Moody M. Panerai R. B. Eames P. J. Potter J. F. 2005 Cerebral and systemic hemodynamic changes during cognitive and motor activation paradigms. Am. J. Physiol.‐Regul. Integr. Comp. Physiol.; 288:R1581-R1588. 15677522 10.1152/ajpregu.00837.2004

[b21] Nakagawa K. Serrador J. M. LaRose S. L. Moslehi F. Lipsitz L. A. Sorond F. A. 2009 Autoregulation in the posterior circulation is altered by the metabolic state of the visual cortex. Stroke; 40:2062-2067. 19359628 10.1161/STROKEAHA.108.545285 PMC2697560

[b22] Nogueira R. Bor‐Seng‐Shu E. Santos M. Negrão C. Teixeira M. Panerai R. B. 2013 Dynamic cerebral autoregulation changes during sub‐maximal handgrip maneuver. PLoS ONE; 8:e70821 23967113 10.1371/journal.pone.0070821 PMC3743835

[b23] Ogoh S. Dalsgaard M. K. Yoshiga C. C. Dawson E. A. Keller D. M. Raven P. B. 2005 Dynamic cerebral autoregulation during exhaustive exercise in humans. Am. J. Physiol.‐Heart Circul. Physiol.; 288:H1461-H1467. 10.1152/ajpheart.00948.2004 15498819

[b24] Oldfield R. 1971 The assessment and analysis of handedness: the Edinburgh inventory. Neuropsychologia; 9:97-113. 5146491 10.1016/0028-3932(71)90067-4

[b25] Panerai R. B. Deverson S. T. Mahony P. Hayes P. Evans D. H. 1999 Effect of CO_2_ on dynamic cerebral autoregulation measurement. Physiol. Meas.; 20:265-275. 10475580 10.1088/0967-3334/20/3/304

[b26] Panerai R. B. Simpson D. M. Deverson S. T. Mahony P. Hayes P. Evans D. H. 2000 Multivariate dynamic analysis of cerebral blood flow regulation in humans. IEEE Bio. Med. Eng.; 47:419-423. 10.1109/10.827312 10743786

[b27] Panerai R. B. Dawson S. L. Eames P. J. Potter J. F. 2001 Cerebral blood flow velocity response to induced and spontaneous sudden changes in arterial blood pressure. Am. J. Physiol. Heart Circ. Physiol.; 280:H2162-H2174. 11299218 10.1152/ajpheart.2001.280.5.H2162

[b28] Panerai R. B. Eames P. J. Potter J. F. 2003 Variability of time‐domain indices of dynamic cerebral autoregulation. Physiol. Meas.; 24:367-381. 12812422 10.1088/0967-3334/24/2/312

[b29] Panerai R. B. Moody M. Eames P. J. Potter J. F. 2005 Cerebral blood flow velocity during mental activation: interpretation with different models of the passive pressure‐velocity relationship. J. Appl. Physiol.; 99:2352-2362. 16099892 10.1152/japplphysiol.00631.2005

[b30] Panerai R. B. Dineen N. E. Brodie F. G. Robinson T. G. 2010 Spontaneous fluctuations in cerebral blood flow regulation: contribution of PaCO_2_. J. Appl. Physiol.; 109:1860-1868. 20884837 10.1152/japplphysiol.00857.2010

[b31] Panerai R. B. Eyre M. Potter J. F. 2012a Multivariate modeling of cognitive‐motor stimulation on neurovascular coupling: transcranial Doppler used to characterize myogenic and metabolic influences. Am. J. Physiol. Regul. Integr. Comp. Physiol.; 303:R395-R407. 22718807 10.1152/ajpregu.00161.2012

[b32] Panerai R. B. Salinet A. S. M. Robinson T. G. 2012b Contribution of arterial blood pressure and PaCO_2_ to the cerebrovascular responses to motor stimulation. Am. J. Physiol. Heart Circ. Physiol.; 302:H459-H466. 22058160 10.1152/ajpheart.00890.2011

[b33] Peebles K. C. Ball O. G. MacRae B. A. Horsman H. M. Tzeng Y. C. 2012 Sympathetic regulation of the human cerebrovascular response to carbon dioxide. J. Appl. Physiol.; 113:700-706. 22744970 10.1152/japplphysiol.00614.2012

[b34] Poulin M. J. Liang P. J. Robbins P. A. 1996 Dynamics of the cerebral blood flow response to step changes in end‐tidal PCO_2_ and PO_2_ in humans. J. Appl. Physiol.; 81:1084-1095. 8889738 10.1152/jappl.1996.81.3.1084

[b35] Rosengarten B. Spiller A. Aldinger C. Kaps M. 2003 Control system analysis of visually evoked blood flow regulation in humans under normocapnia and hypercapnia. Eur. J. Ultrasound; 16:169-175. 12573785 10.1016/s0929-8266(02)00070-8

[b36] Salinet A. S. M. Panerai R. B. Robinson T. G. 2012a Effects of active, passive and motor imagery paradigms on cerebral and peripheral hemodynamics in older volunteers: a functional TCD study. Ultrasound Med. Biol.; 38:997-1003. 22502887 10.1016/j.ultrasmedbio.2012.02.016

[b37] Salinet A. S. M. Robinson T. G. Panerai R. B. 2012b Reproducibility of cerebral and peripheral haemodynamic responses to active, passive and motor imagery paradigms in older healthy volunteers: A fTCD study. J. Neurosci. Methods; 206:143-150. 22366252 10.1016/j.jneumeth.2012.02.011

[b38] Salinet A. S. M. Robinson T. G. Panerai R. B. 2013a Active, passive and motor imagery paradigms: component analysis to assess neurovascular coupling. J. Appl. Physiol.; 114:1406-1412. 23449939 10.1152/japplphysiol.01448.2012

[b39] Salinet A. S. M. Robinson T. G. Panerai R. B. 2013b Cerebral blood flow response to neural activation after acute ischaemic stroke: a failure of myogenic regulation? J. Neurol.; 260:2588-2595. 23824356 10.1007/s00415-013-7022-z

[b40] Serrador J. M. Picot P. A. Rutt B. K. Shoemaker J. K. Bondar R. L. 2000 MRI measures of middle cerebral artery diameter in conscious humans during simulated orthostasis. Stroke; 31:1672-1678. 10884472 10.1161/01.str.31.7.1672

[b41] Spronck B. Martens E. G. H. J. Gommer E. D. van de Vosse F. N. 2012 A lumped parameter model of cerebral blood flow control combining cerebral autoregulation and neurovascular coupling. Am. J. Physiol. Heart Circ. Physiol.; 303:H1143-H1153. 22777421 10.1152/ajpheart.00303.2012

[b42] Szabo K. Lako E. Juhasz T. Rosengarten B. Csiba L. Olah L. 2011 Hypocapnia induced vasoconstriction significantly inhibits the neurovascular coupling in humans. J. Neurol. Sci.; 309:58-62. 21831399 10.1016/j.jns.2011.07.026

[b43] Tiecks F. P. Lam A. M. Aaslid R. Newell D. W. 1995 Comparison of static and dynamic cerebral autoregulation measurements. Stroke; 26:1014-1019. 7762016 10.1161/01.str.26.6.1014

[b44] Vernieri F. Pasqualetti P. Passarelli F. Rossini P. M. Silvestrini M. 1999 Outcome of carotid artery occlusion is predicted by cerebrovascular reactivity. Stroke; 30:593-598. 10066857 10.1161/01.str.30.3.593

[b45] Willie C. K. MacLeod D. B. Shaw A. D. Smith K. J. Tzeng Y. C. Eves N. D. 2012 Regional brain blood flow in man during acute changes in arterial blood gases. J. Physiol.; 590:3261-3275. 22495584 10.1113/jphysiol.2012.228551 PMC3459041

[b46] Xu F. Uh J. Brier M. R. Hart J. Jr Yezhuvath U. S. Gu H. 2011 The influence of carbon dioxide on brain activity and metabolism in conscious humans. J. Cereb. Blood Flow Metab.; 31:58-67. 20842164 10.1038/jcbfm.2010.153 PMC3049465

[b47] Yoon S. Zuccarello M. Rapoport R. M. 2012 pCO_2_ and pH regulation of cerebral blood flow. Front. Physiol.; 3:365 23049512 10.3389/fphys.2012.00365 PMC3442265

[b48] Zhang R. Zuckerman J. H. Giller C. A. Levine B. D. 1998 Transfer function analysis of dynamic cerebral autoregulation in humans. Am. J. Physiol.; 274:H233-H241. 9458872 10.1152/ajpheart.1998.274.1.h233

